# Effect of Metformin on Serum Ferritin Level in Women with Polycystic Ovary Syndrome

**Published:** 2011-07-01

**Authors:** S Behradmanesh, Gh H Ranjbar Omrani, F Ghazanfarpour, A Baradaran

**Affiliations:** 1 Department of Internal Medicine and Endocrinology, Shahr-e-Kord University of Medical Sciences, Shahr-e-Kord, Iran; 2 Department of Internal Medicine and Endocrinology, Endocrine and Metabolic Re-search Center, Nemazee Hospital, Shiraz University of Medical Sciences, Shiraz, Iran; 3 Skin Diseases and Leishmaniasis Research Center, Isfahan University of Medical Sciences, Isfahan, Iran; 4 Department of Pathology, Isfahan University of Medical Sciences, Isfahan, Iran

**Keywords:** Ferritin, Polycystic Ovary Syndrome, Metformin

## Abstract

**Background:**

Polycystic ovary syndrome (PCOS) is one of the most common diseases among women associat-ed with various inflammatory reactants such as C-reactive protein (CRP) and ferritin. This study aimed to investi-gate the effect of metformin on probable reduction of serum ferritin in patients with PCOS.

**Methods:**

This study was conducted on 45 patients with PCOS who had not other systemic diseases and did not take any medications. Weight, waist and hip circumstances (WHR), body mass index (BMI), metabolic indexes, CRP, ferritin and “Homeostasis Model Assessment of Insulin Resistance (HOMA-IR) ˝ were measured before the study. Metformin (500 mg/tid) tablets were prescribed for three months and then same above parameters were re-measured.

**Results:**

Of 45 patients, 19 (42.2%) were overweight and 14 (31.1%) were obese. After drug therapy, there was a significant reduction in waist circumstance and serum ferritin. This reduction was significant only in the lean and overweight groups but not in the obese group. There was not significant association between serum ferritin and CRP, HOMA-IR, BMI and WHR. There was not significant correlation between CRP and HOMA-IR and also BMI.

**Conclusion:**

The effect of metformin on reduction of serum ferritin was not significant just in obese group and was not associated with metabolic and anthropometric indexes.

## Introduction

Polycystic ovary syndrome (PCOS) is one of the most common diseases with prevalence of 6-7% among women in their reproductive age.[1][2][3][4] Obesity, hyper-androgenism, ovarian dysfunction and insulin re-sistance are features of this disease.[5] In addition, other diseases such as type 2 diabetes mellitus (T2DM), metabolic syndrome (MetS) and cardiovascular dis-eases (CVD) are related to this syndrome.[6][7][8][9] It has been recently found that acute phase reactants of in-flammation including C-reactive protein (CRP) are higher than normal in T2DM, hyperinsulinemia[10] and PCOS.[11] Serum level of CRP has a higher value in obese PCOS subjects than those non-obese with PCOS. A treatment with metformin for six months lowers CRP serum level in all subjects as well as obese and non–obese.[12] Another acute phase reactants of inflammation is ferritin which is higher than nor-mal in patients with metabolic syndrome, T2DM pa-tients and PCOS.[13][14][15] Serum ferritin levels in over-weight and obese women with PCOS are significantly higher compared to overweight and obese women without this disorder.[15][16][17][18]

Metformin causes improvement of insulin re-sistance in patients with PCOS.19,20 Regarding the prevalence of PCOS among women at reproductive

age4 and accompany of acute phase reactants of in-flammation with the risk of cardiovascular diseases,21 we sought to investigate the effect of metformin on lowering serum ferritin level in PCOS, obese and non-obese patients.

## Materials and Methods

This is a before-after quasi experimental study on 45 PCOS women. The PCOS subjects were selected based on Homburg criteria and others.[12][22] The study was carried out in clinics related to Shiraz University of Medical Sciences, Shiraz Iran, from January 2006 and lasted for six months.

Exclusion criteria were pregnancy, diabetes mellitus, psychotic disorders, hypertension, cardiovascular dis-eases, smoking, alcohol drinking and oral contraceptive pill taking, anti-inflammatory drugs and any other medi-cation for hyperlipidemia, hypertension, and glucose intolerance in the past six months were also the exclu-sion criteria. Patients who were also on special diet or on standard sports to lose weight, all were excluded too. The secondary PCOS was also ruled out.

All subjects signed the consent forms and the Ethics Committee of the university approved the study. Basic measurements on anthropometric indexes included waist and hip (cm) (waist was calculated by the great-est circumstance between the last rib and iliac crest and the hip was calculated by the greatest circumstance from both greater femoral trochanters),[23] height (m), weight (kg), BMI (kg/m2) and WHR.

BMI≤25 was considered as normal, 25<BMI<30 overweight and BMI≥30 was considered as obese. WHR>0.8 was considered as a sign of central obesi-ty.[24]In addition, fasting insulin was measured with immunotech kit (made in Check by IRMA method), while fasting plasma glucose (FPG), triglycer-ide(Tg), total cholesterol (chol), HDL and LDL [through enzymatic calorimetric method], serum fer-ritin and high sensitive C reactive protein (hsCRP) [by IRMA method] levels that were measured using standard kits.

Homeostasis Model Assessment of Insulin Re-sistance (HOMA-IR) was calculated by fasting serum insulin (pmol/L) multiplied by FBS (mmol/L) divided to 22.5.[25] Blood pressure was measured by standard method too. In the first week, apo-metformin (Apotex Inc., Toronto, Canada) was prescribed (500 mg/bid) to lower gastrointestinal complications and then reached 500 mg/tid and continued for 12 weeks. The subjects were asked to have a normal diet (30-35 kcal /kg/day) together with ordinary physical activity. At the end of three months (12 weeks), anthropometric indexes were re-measured and recorded. The above-mentioned lab tests were repeated. Insulin resistance was calculated and systolic diastolic BP was re-measured by the same method too.

Data were expressed as mean±SD and frequency distribution. Normal distribution of the data was checked with Kolmogrov Smirnov test. Paired t-test and ANOVA by Tukey posthoc were used to investi-gate the changes in variables before and after treat-ment as well as in subgroups. Correlation among some variables was done by Pearson's bivariate corre-lation coefficient test. Data were analyzed by SPSS software (version 13.0, Chicago, IL, USA). A P-value<0.05 was considered significant.

## Results

The age of the subjects was 22.5±4.5 years. Of 45 subjects, 12 (26.7%) had normal BMI, 19 (42.2%) were overweight and 14 (31.1%) were obese. Forty four (97.8%) had central obesity based on WHR. Comparison of the changes in anthropometric indexes before and after three months with metformin showed that hip circumference had a significant decrease while other parameters showed no significant differ-ence (Table 1).

**Table 1:** Comparison of the changes in anthropometric indexes before and after three months treatment (n=45).

	**Before**	**After 3-months**	**P value**
Weight (kg)	70.9±12.2	70.2±11.9	0.08
BMI (kg/m^2^)	27.7±4.1	27.4±4.2	0.1
Waist (cm)	96.9±11	95.8±11.4	0.2
Hip (cm)	105±7.3	104±7.3	0.03
WHR	0.92±0.06	0.91±0.07	0.7
Systolic BP (mmHg)	112.3±8.5	113.3±8.1	0.4
Diastolic BP (mmHg)	72.1±8.2	74.4±7.5	0.09

Table 2 shows the comparison of metabolic parameters. Serum ferritin level had a significant decrease (p<0.001, t=4.42). Comparison of basic and after three months treatment of hsCPR, ferritin and HOMA-IR with metformin showed that basic serum ferritin was not different between groups (normal, overweight and obese) (Not Significant). Comparison of serum ferritin , hsCRP and HOMA-IR before and after three months treatment with metformin among mentioned above sub-groups of BMI showed a significant difference of HOMA-IR (p=0.04), serum ferritin (p=0.02) in normal BMI group, while there was not significant difference of hsCRP (p=0.4). In overweight group, there was only significant difference of serum ferritin (p=0.001). There was not significant difference of parameters before and after treatment in obese sub-group.

**Table 2:** comparison of the changes in metabolic parameters before and three months after treatment (n=45)^a^.

	**Before**	**After 3-months**	**P value**
Insulin (μU/mL)	11.8±9.6	11.6±8.3	0.9
FPG (mg/dL)	77.3±8.4	79.6±15.1	0.3
TG (mg/dL)	156.3±76.5	157.5±78.3	0.4
Total cholesterol (mg/dL)	191.8±38.5	193.8±40	0.5
LDL	90.3±31.4	90.3±28.3	0.3
HDL	69.8±19.5	71.6±15	0.5
Ferritin (μg/L)	68.5±51.3	35.1±33.9	0.00
hs CRP (mg/L)	3.2±3.7	2.3±2.2	0.05

^a^ FPG, TG and hsCRP denotes fasting plasma glucose, triglyceride and highly sensitive CRP respectively.

Data analysis by ANOVA showed no significant difference of CRP (p=0.8), ferritin (p=0.1) and HOMA-IR (p=0.07) among three groups of obese, non-obese and overweight subjects.

Pearson's correlation test showed an association between CRP and insulin resistance indexes before intervention (p=0.02, r=0.34) (Table 3). There was not association between serum ferritin with CRP and insulin resistance either before or after three months of treatment (p>0.05).Associations between CRP, HOMA-IR with BMI and WHR before and after the treatment has been presented in Table 4. There was not association between ferritin and these anthropometric indexes either before or three months after treatment (p>0.05).

**Table 3:** Mean±SD of CRP, ferritin and insulin resistance indexes in three groups of obese, overweight and lean before and after treatment.

	**Obese (n=14)**		**Overweight (n=19)**		**Lean (n=12)**	
	**Before**	** After3 months**	**Before**	**After3 months**	**Before**	**After3 months**
hs CRP	5.07±5.7	2.9±1.9	2.8±2.4	2.5±2.8	1.4±0.73	1.3±0.38
Ferritin	77.6±43	59.3±48.7	52.3±24.8	23.7±13.5	83.5±80.9	24.8±20.7
HOMA-IR	2.9±1.3	3.5±2.4	1.8±0.9	2.5±1.2	1.3±0.87	0.8±0.25

**Table 4:** Association of CRP, HOMA-IR with WHR and BMI before and after treatment.

	**CRP**		**Homa-IR**	
	**Before**	**After3 months**	**Before**	**After 3 months**
BMI				
r	0.33	0.39	0.43	0.54
p	0.02	0.008	0.003	0.00
WHR				
r	0.23	0.2	0.14	0.41
p	0.12	0.09	0.35	0.005

## Discussion

In this study, we found that three months intake of metformin in PCOS patients, irrespective to BMI groups, caused a reduction in serum ferritin. In a comparison between two groups of obese PCOS, reduction of serum ferritin during treatment with metformin and diane was investigated by Luque-Ramírez et al. [18] They showed that taking metformin for three months and also six months resulted in a reduction in serum ferritin among these patients. After data analysis based on BMI, they found that intake of metformin for three months reduced ferritin just in overweight and lean groups but this reduction was not significant in the obese group. They concluded that the reason for this difference in obese group can possibly be due to the comparison of metformin with diane.[18] To investigate the reduction of CRP in PCOS during metformin treatment, Morin-Papunen et al. showed that CRP significantly decreased after three months and 6 months post treatment respectively compared to the beginning of the study. These finding was not in accordance with our findings. In the present study, there was not significant reduction in any of the subgroups possibly due to the sample size compared to our findings.[12]

Previous studies showed that obese PCOS had higher insulin resistance compared to normal weight PCOS.26,27 Insulin resistance and hyperinsulinemia resulted into an increase in iron storage through bowel absorption, as indicated in metabolic syndrome and T2D.17,28,29 It was thought that metformin with respect to its effect on central obesity and facilitating insulin function30 reduces insulin sensitivity index together with a decrease in serum ferritin that does not affect CRP in comparison to the obese PCOS taking diane.[17] In the present study, there was not any association between BMI, WHR, HOMA-IR and serum ferritin not only before but also after treatment possibly due to lack of control group or sample size difference in these two studies (19 vs 45). While in the present study, there was not any association between CRP and ferritin either before or after the treatment, former studies showed that the increase in ferritin is independent to the increase in CRP.15,18 Previous studies showed an increase in CRP serum level in obese people31 and an association between abdominal obesity, insulin resistance and CRP.32 It is was found that anthropometric parameters especially waist and WHR are modified with metformin in PCOS resulting in a decrease in CRP.[12]Although, there was not a significant decrease in CRP with metformin among all subjects and in subgroups in the present study, there was a direct significant association between CRP with BMI (before and after treatment). There was not a significant decrease in HOMA-IR in all subjects while a significant decrease was noticed among subgroups.

Among other anthropometric indexes and metabolic parameters of our study, hip circumference showed a significant reduction, although among others a decrease was seen but was not significant. In an another study in obese PCOS patients conducted by Morin-Papunen et al., intake of metformin for three straight months showed no significant decrease for BMI, WHR and insulin sensitivity index whereas a significant decrease was observed for WHR after six months. In addition, FPG and fasting insulin showed a significant reduction after three months.[33]The effect of metformin on non-obese PCOS was also investigated in an another study by Morin-Papunen et al. They found a significant reduction in BMI, WHR, FPG and fasting insulin after three months of therapy with metformin, while the reduction in insulin sensitivity index was significant. The different results of two above studies compared to our findings was the existence of a control group and low sample size.[33] There was not a significant difference between our findings and those two above regarding the HOMAIR and the reduction of anthropometric indexes especially in non-obese subjects. To investigate the basic CRP, ferritin and HOMA-IR in the related subgroups, there was a significant difference in CRP and HOMA-IR, in obese patients compared to normal weights (p=0.03), something consistent with former studies.[12]The level of ferritin was not different in three groups. In a comparative research serum ferritin in PCOS group (compared to healthy group according to identical weight) serum ferritin was found not to be different in those with normal weight, while, mean serum ferritin level in overweight and obese PCOS subjects was significantly higher compared to the control group with identical weight, apparently not consistent with the present study possibly due to lack of a control group.[12] In this study, we can concluded that intake of metformin for three months causes a reduction in serum ferritin in all PCOS subjects. This reduction was independent of subjects' weight group, CRP and HOAM-IR. Although hyperinsulinemia was investigated as an iron loading agent in PCOS obese subjects in former studies,18 regarding the results of the present study, the reason for iron overload still needs further consideration by other investigators.
